# Foreign body aspiration in an adult: An endobronchial “Melon-oma”

**DOI:** 10.5339/qmj.2022.fqac.23

**Published:** 2022-04-04

**Authors:** Irfan Ul Haq, Mansoor Hameed, Shakeel Ahmed, Mousa Hussein

**Affiliations:** ^1^Pulmonary Division, Department of Medicine, Hamad Medical Corporation E-mail: IHaq@hamad.qa

**Keywords:** adult, FB aspiration, bronchoscopy

## Abstract

**Background:** Foreign body (FB) aspirations in adults are relatively uncommon. The most commonly aspirated FBs in adults are organic, especially vegetable matter, peanuts, and fragments of bones. We report a rare case of a FB discovered in the left main bronchus of an adult male admitted to the intensive care unit. **Case report:** A 52-year-old male smoker with COPD presented to the emergency department with a two-day history of increasing dyspnea and cough. He was hypoxic and febrile with a temperature of 38°C. Auscultation revealed decreased breath sounds over the left lung and a few rhonchi on the right side. Chest x-ray showed left lung collapse. His condition rapidly worsened, and he was immediately intubated for acute respiratory failure. CT chest identified a large endobronchial mass obstructing the left main bronchus. Flexible bronchoscopy confirmed a soft and mobile brownish lesion in the left main bronchus. The histological appearance of the specimen retrieved was consistent with an organic foreign body. This was later identified as a melon chunk. It was removed successfully via flexible bronchoscopy by cutting it into smaller pieces to aid retrieval. **Conclusion:** FB aspiration can occur in all age groups but is less common in adults accounting for only 0.16%–0.33% of adult bronchoscopic procedures. Early detection of an aspirated FB is essential to avoid significant complications, morbidity, and mortality.

## Figures and Tables

**Figure 1.**
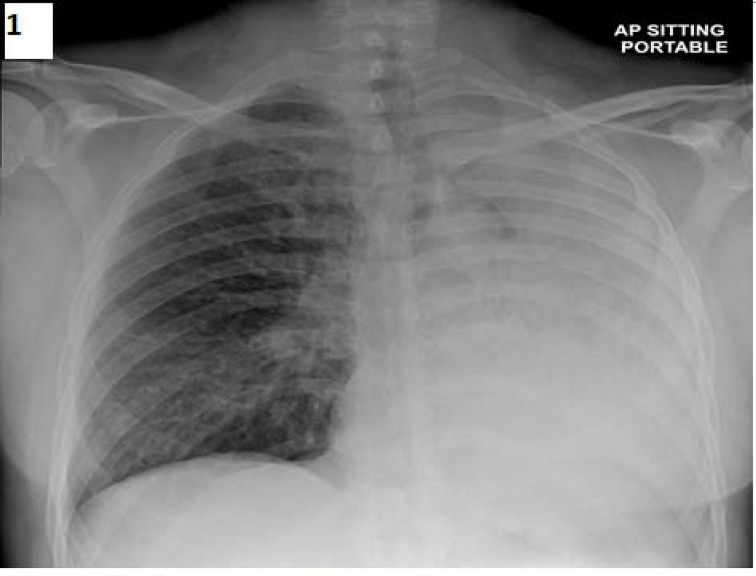
Chest x-ray showing left lung collapse

**Figure 2.**
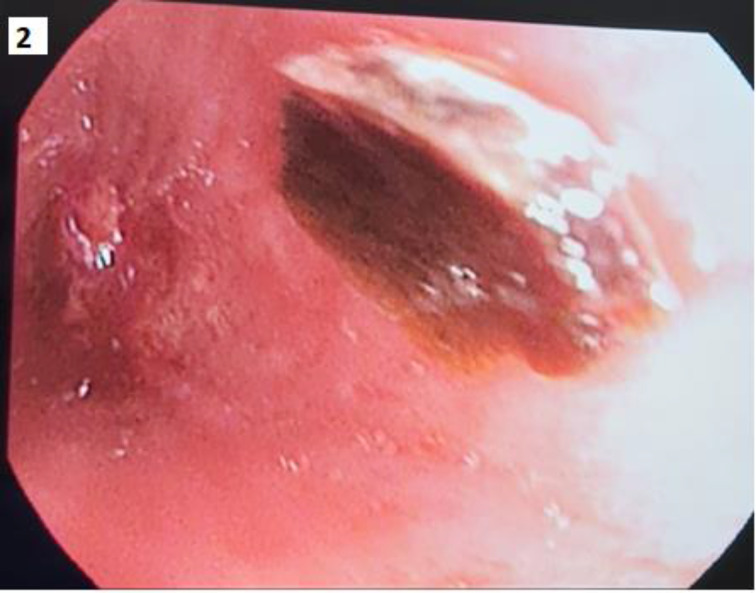
Bronchoscopy image showing foreign body (melon chunk) in the airway.

